# Evidence for a protective role for the rs805305 single nucleotide polymorphism of dimethylarginine dimethylaminohydrolase 2 (DDAH2) in septic shock through the regulation of DDAH activity

**DOI:** 10.1186/s13054-018-2277-5

**Published:** 2018-12-11

**Authors:** Simon Lambden, James Tomlinson, Sophie Piper, Anthony C. Gordon, James Leiper

**Affiliations:** 1 Department of Medicine, University of Cambridge, Addenbrooke’s Hospital, Cambridge, CB2OQQ UK; 2 Institute of Cardiovascular and Medical Sciences, University of Glasgow, University Avenue, Glasgow, G12 8QQ UK; 3 MRC London Institute of Medical Sciences, Hammersmith Hospital Campus, Du Cane Road, London, W12 0NN UK; 4 Section of Anaesthetics, Pain Medicine and Intensive Care, Faculty of Medicine, Imperial College London, London, UK

**Keywords:** Mortality/survival, Clinical studies, Translational studies, Genetics, Septic shock, Nitric oxide, Nitric oxide synthase, Sepsis

## Abstract

**Background:**

Dimethylarginine dimethylaminohydrolase 2 (DDAH2) regulates the synthesis of nitric oxide (NO) through the metabolism of the endogenous inhibitor of nitric oxide synthase, asymmetric dimethylarginine (ADMA). Pilot studies have associated the rs805305 SNP of DDAH2 with ADMA concentrations in sepsis. This study explored the impact of the rs805305 polymorphism on DDAH activity and outcome in septic shock.

**Methods:**

We undertook a secondary analysis of data and samples collected during the Vasopressin versus noradrenaline as initial therapy in septic shock (VANISH) trial. Plasma and DNA samples isolated from 286 patients recruited into the VANISH trial were analysed. Concentrations of L-Arginine and the methylarginines ADMA and symmetric dimethylarginine (SDMA) were determined from plasma samples. Whole blood and buffy-coat samples were genotyped for polymorphisms of DDAH2. Clinical data collected during the study were used to explore the relationship between circulating methylarginines, genotype and outcome.

**Results:**

Peak ADMA concentration over the study period was associated with a hazard ratio for death at 28 days of 3.3 (95% CI 2.0–5.4), *p* < 0.001. Reduced DDAH activity measured by an elevated ADMA:SDMA ratio was associated with a reduced risk of death in septic shock (*p* = 0.03). The rs805305 polymorphism of DDAH2 was associated with reduced DDAH activity (*p* = 0.004) and 28-day mortality (*p* = 0.02). Mean SOFA score and shock duration were also reduced in the less common G:G genotype compared to heterozygotes and C:C genotype patients (*p* = 0.04 and p = 0.02, respectively).

**Conclusions:**

Plasma ADMA is a biomarker of outcome in septic shock, and reduced DDAH activity is associated with a protective effect. The polymorphism rs805305 SNP is associated with reduced mortality, which is potentially mediated by reduced DDAH2 activity.

**Trial registration:**

ISRCTN Registry, ISRCTN20769191. Registered on 20 September 2012.

**Electronic supplementary material:**

The online version of this article (10.1186/s13054-018-2277-5) contains supplementary material, which is available to authorized users.

## Background

Sepsis arises when an infective organism provokes a series of host responses, the dysregulation of which leads to organ dysfunction. There is a spectrum of disease which, at its most severe, is characterised by cellular, tissue, vascular and cardiac abnormalities [1, 2]. Collectively these pathological changes lead to altered cellular metabolic function, impaired tissue oxygen availability and reduced utilisation by the tissues [3–6]. Sepsis is a major health challenge in both the developing and the developed world, with more than 2 million cases in the USA [7] and an estimated 5.3 million deaths worldwide [8] each year.

Nitric oxide (NO) is a critical mediator of the vascular, immune and cardiac response to infection [3, 9–12]. NO is synthesised by the three isoforms of nitric oxide synthase (NOS) in a process that is tightly regulated at every stage from gene expression to substrate and co-factor availability [13]. The discovery of a family of arginine derivatives, known as the methylarginines (MA), which act as inhibitors by competing with the binding of L-arginine to the active site of the NOS enzyme, is one of few examples where an enzyme system is regulated by endogenous competitive inhibition [14].

**Fig. 1:**
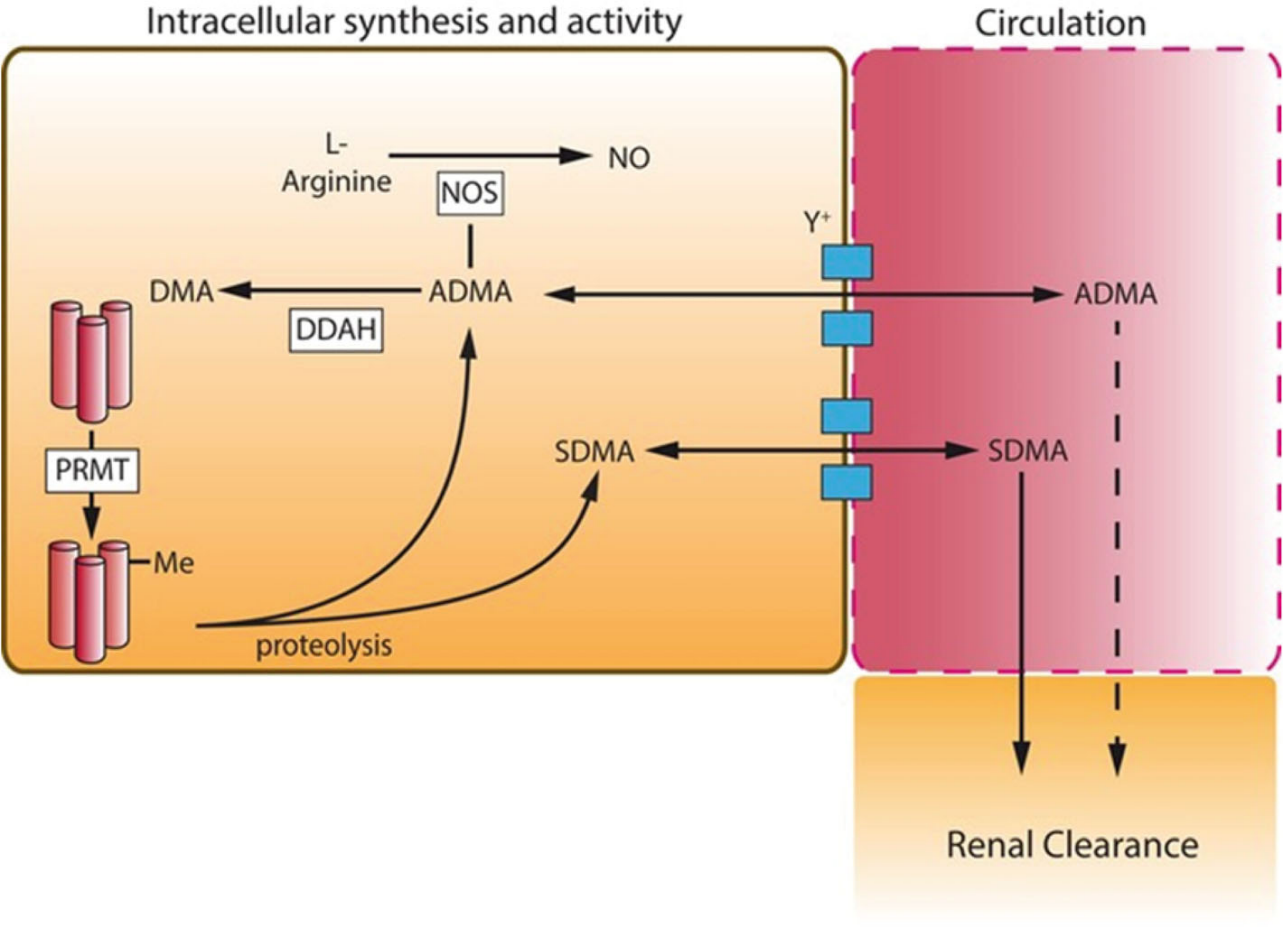
Representative image of the synthesis and regulation of asymmetric dimethylarginine (ADMA) and symmetric dimethylarginine (SDMA) in sepsis. Protein arginine methyl transferases (PRMT) catalyse the methylation of arginine containing protein residues to ADMA and SDMA, which are released upon proteolysis. ADMA and SDMA are transported via the y^+^ cationic amino acid transporter into and out of the circulation. SDMA is not metabolised in most cell,s whereas ADMA is metabolised by the two isoforms of dimethylarginine dimethylaminohydrolase (DDAH) in a wide range of tissues. ADMA acts intracellularly to inhibit nitric oxide synthase (NOS), SDMA has no action on NOS isoforms. SDMA is cleared by the kidney largely unchanged and in small part through metabolism by AGXT2 (not shown). ADMA is largely metabolised by DDAH to dimethylamine (DMA); a small amount is cleared unchanged through the kidney. In sepsis, the synthesis of ADMA and SDMA may be increased through high protein-turnover in patients in a catabolic state and reduced renal clearance as a consequence of acute kidney injury. Differences in the relative concentrations of these methylarginines reflect their differential metabolism by DDAH isoforms as only ADMA is a DDAH substrate. Note, monomethylarginine (L-NMMA) is considered to have the same synthetic pathway, activity, metabolism and clearance as ADMA but is present in only 10% of the concentration

Three MA subtypes have been identified in mammals, asymmetric dimethylarginine (ADMA), symmetric dimethylarginine (SDMA) and monomethylarginine (L-NMMA) [15]. Within the cell, both ADMA and L-NMMA act through competitive inhibition of L-arginine binding to the NOS enzymes (Fig. 1). L-NMMA and ADMA are equipotent but L-NMMA is present at only around 10% of the concentration of ADMA in most tissues and so plays a lesser role in the regulation of NOS activity [16]. By contrast, SDMA does not compete with L-arginine at physiological and pathophysiological concentrations and has no apparent impact on NOS activity. Elevation of plasma ADMA concentration has been associated with risk in cardiac [17] and chronic renal disease [18]. In sepsis, a number of studies in heterogeneous clinical populations have suggested that plasma ADMA concentration is associated with illness severity and death [19–23].

Within the cell, ADMA is tightly regulated by the two isoforms of dimethylarginine dimethylaminohydrolase (DDAH1 and DDAH2) that hydrolyse the two active methylarginines to methylamines and L-citrulline [24, 25]. Only around 10% of ADMA is cleared by the kidney in normal conditions [26]. In contrast, SDMA is not metabolised by DDAH and is largely cleared by the kidney. The two isoforms of DDAH have similar structure but arise from different genetic loci and are expressed in differing tissue distributions [24]. SNPs of the *DDAH2* gene have been associated with disease severity and/or progression in a number of disease states including hypertension [27], myocardial infarction [28] and chronic renal failure [29].

The production of NO in sepsis is regulated by a number of different processes in addition to the level of expression of NOS isoforms. These include changes in substrate and co-factor bioavailability, and cellular concentrations of ADMA, which competes with L-arginine binding to the NOS isoforms to regulate NO production. ADMA concentrations are regulated by the DDAH enzymes and their expression in different tissues may suggest differential roles in regulating the inflammatory response. The *DDAH2* gene is located in the major histocompatibility complex (MHC)III region of chromosome 6 and DDAH2 is the only isoform found in immune cells [24]; DDAH1 by contrast, the gene that is found on chromosome 1, is an important regulator of vascular function and has been shown to modulate the haemodynamic response in animal models of sepsis [30, 31]. These observations have led to the suggestion that DDAH2-mediated regulation of ADMA concentration may play a role in NO mediated inflammatory conditions [32] such as septic shock.

We undertook a secondary analysis of samples and clinical data from the Vasopressin versus noradrenaline as initial therapy in septic shock (VANISH) trial, which was a randomised controlled trial of two vasoactive treatments in septic shock. Our study tested the hypothesis that ADMA has a mechanistic role in determining outcome from septic shock and that the polymorphism of the DDAH2 promoter (rs805305) may impact on methylarginine metabolism and outcome in these patients.

## Methods

In the VANISH trial, adult patients with septic shock, from 18 centres in the UK were recruited within a maximum of 6 h after the onset of hypotension to participate in a double-blind randomised controlled trial of vasopressin versus norepinephrine as the first-line treatment of vasopressor-dependent septic shock. The study was a 2 × 2 design with patients in each group randomised to receive placebo or hydrocortisone in addition to their primary intervention drug in the event that they did not respond adequately to study drug monotherapy. The study received ethics committee approval (Oxford A research ethics committee (12/SC/0014)) and was registered prior to the start of recruitment (ISRCTN20769191). Extensive clinical data were collected at baseline and on a daily basis up to day 28 after randomisation. The detailed protocol for this study and the primary outcome has been published previously [33, 34].

In 16 of the participating centres, local research teams were able to collect serial blood samples for subsequent analysis. Samples were collected at up to four time points during the study, after randomisation but prior to starting study drug (time point 0), and on study days 3, 5 and 7 thereafter.

Whole blood was collected in a 10-ml EDTA tube and immediately stored on ice. Within 15 min of sampling, the tube underwent centrifugation at 1000 g for 10 min. At the end of this period, plasma and buffy-coat aliquots were collected in separate cryotubes, labelled appropriately and immediately stored at − 80 °C. If staffing levels out of hours prevented sample processing, whole blood was stored for subsequent DNA analysis only.

Samples were analysed *en bloc* at the end of the study period. Methylarginine and L-arginine concentrations were analysed using an established high-performance liquid chromatography mass spectrometry approach [30, 35, 36]. In brief, samples were defrosted on ice and a 100-μL aliquot of plasma was collected and a known concentration of deuterium-labelled [7] ADMA standard was added. Following protein precipitation with methanol, the solution underwent centrifugation at 16000 rcf for 10 min. The supernatant was then dried and the residue re-suspended in mobile phase (0.1% formic acid) for analysis. A standard curve of ADMA, SDMA and L-arginine samples of 10 known concentrations was prepared (0–10 μM).

The presence of the rs805305 SNP in the *DDAH2* gene was determined using genomic DNA extraction from buffy coat and whole blood samples by LGC group (Hertfordshire, UK). In a parallel experiment, the genotype of eight polymorphisms of the *DDAH1* gene was also determined (Additional file 1: Table S5). Genotyping was undertaken using a proprietary competitive allele-specific PCR technique known as Kompetitive Allele Specific (KASP) PCR genotyping system which has been extensively used for uniplex SNP analysis [37, 38]. Additional quality-control criteria included interplate and intraplate duplicate testing and clear separation of signal clusters.

Statistical analysis was performed using the Prism software package (GraphPad Inc., CA, USA) and SPSS v25 (IBM plc, USA). Normally distributed data were analysed using the *t* test or analysis of variance (ANOVA) with Bonferroni post-test comparison of groups as appropriate. Non parametric data were analysed using the Mann-Whitney U test to compare two groups or Kruskall-Wallis analysis for multiple groups, with Dunn’s multiple comparison test undertaken to compare differences between the groups. Survival was analysed by the log rank test to determine mortality trends and multivariate Cox regression analysis was undertaken to consider potential for confounding by other clinical factors.

## Results

### Clinical population

Of the 408 patients included in the VANISH trial modified intention-to-treat analysis, 209 had buffy-coat samples and 75 had whole blood stored that was suitable for DNA extraction and genotype analysis. In 249 of those patients, at least one plasma sample was available for analysis of methylarginine and L-arginine concentrations*.* Peak methylarginine concentrations were defined as the highest value measured from available samples for each patient. In patients who did not survive for the full sample collection period, the peak value from the available timepoints was selected. Patients in this subgroup had similar baseline characteristics to those of the VANISH population as a whole and as there was no difference in the primary outcome of renal-failure-free days or mortality between the treatment groups in the VANISH trial, all patients were analysed together in this study (Additional file 1) [34]. In the population that underwent genotyping analysis, median (IQR) duration of shock was 47.5 (26.1–87.5) h.

### Temporal pattern of L-Arginine and methylarginines in septic shock

**Fig. 2:**
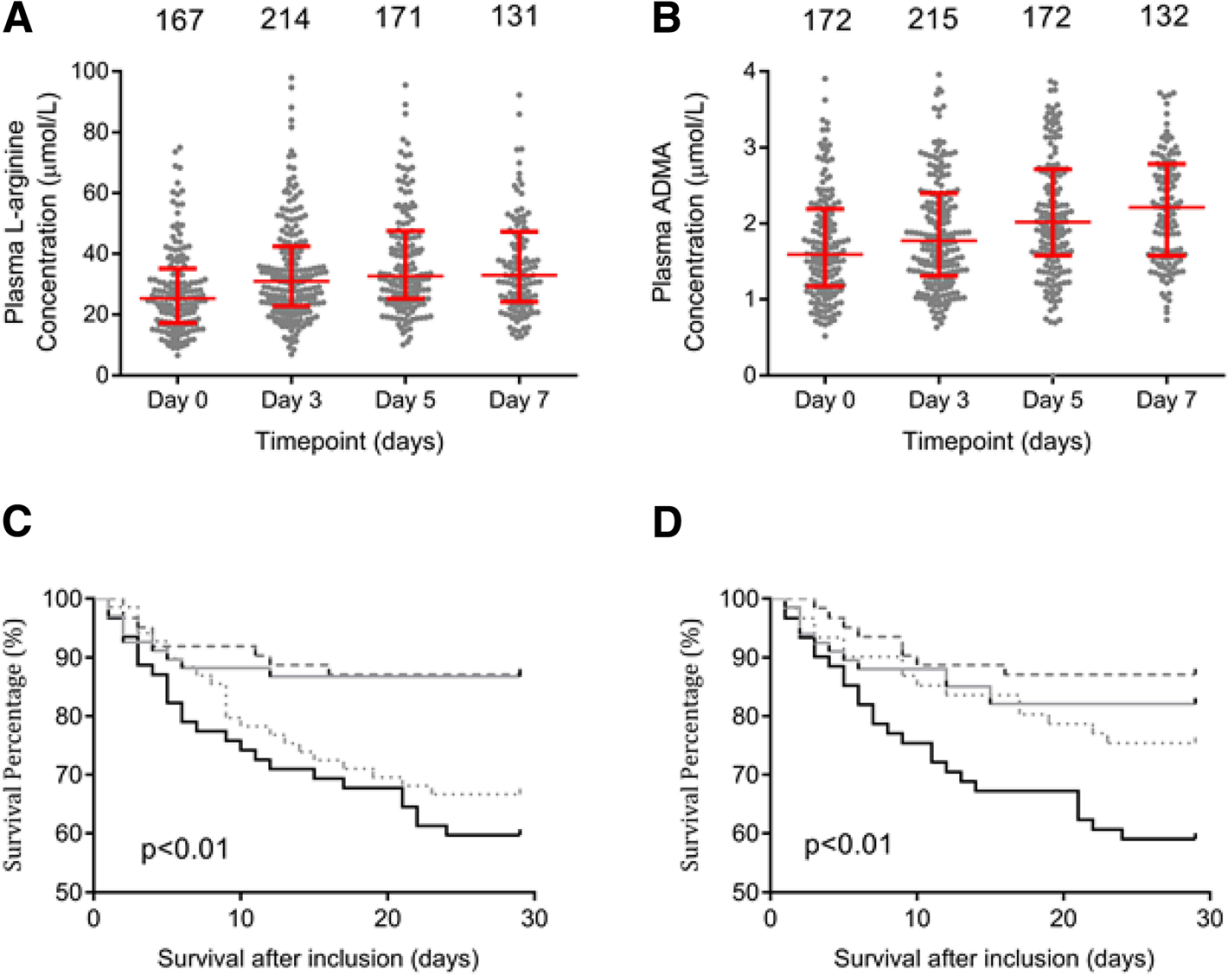
Temporal patterns in L-arginine and methylarginines over time in septic shock. Plasma L-arginine (**a**), asymmetric dimethylarginine (ADMA) (**b**) concentration changes over the first 7 days of inclusion in the VANISH trial; notation describes the number of samples available for analysis at each time point. Median and interquartile range plotted in red (both *p* < 0.001 over the time course (analysis of variance). Kaplan-Meier analysis of the association between methylarginine concentrations and outcome over time in septic shock following inclusion in the VANISH trial. A higher peak plasma ADMA (**c**) and symmetric dimethylarginine (SDMA) (**d**) concentration is associated with reduced survival in patients with septic shock. Data presented in quartiles (black line - top 25%, small-dotted grey line - 2nd quartile, dashed grey line - 3rd quartile, solid grey line - lowest quartile)

Plasma L-arginine and methylarginine concentrations were measured at each available time point and temporal patterns analysed (Table 1). Median L-arginine concentration was at its lowest on study inclusion then rose over subsequent study days (Fig. 2a). ADMA concentrations were elevated over normal plasma values [39] and median plasma ADMA concentration increased over the course of the study period (*p* < 0.001) and was significantly higher on days 5 and 7 than at study inclusion (all *p* < 0.05, (Fig. 2b). No significant changes in plasma SDMA concentration were observed during the study period, *p* = 0.478 (Additional file 1: Figure S1A).

**Table 1:** Pattern of plasma L-arginine, asymmetric dimethylarginine (ADMA) and symmetric dimethylarginine (SDMA) concentrations (μM, median (IQR)) over the first 7 days of the VANISH trial

	Inclusion	Day 3	Day 5	Day7
L-arginine	25.3 (17.2–35.1)	30.0 (22.8–42.4)	32.6 (25.2–47.5)	32.9 (24.2–47.3)
ADMA	1.6 (1.17–2.2)	1.8 (1.3–2.4)	2.0 (1.6–2.7)	2.2 (1.6–2.8)
SDMA	3.4 (2.0–5.2)	3.4 (1.8–5.3)	3.4 (2.0–5.2)	3.1 (1.7–4.8)

The impact of treatment group allocation on L-arginine and methylarginine concentrations was determined in order to rule out any treatment group effect. No differences were detected in plasma L-arginine or methylarginine concentrations across treatment allocations (Additional file 1: Table S1).

### L-arginine and methylarginine concentrations and outcome

Median (IQR) L-arginine concentrations were similar in both survivors and non-survivors at recruitment and on day 3. A pattern of higher L-arginine concentration was, however, associated with non-survivors on days 5 and 7 (Additional file 1: Table S2, Figure S1B).

Plasma concentrations of ADMA and SDMA were elevated at each time point in patients who died compared to survivors (Additional file 1: Table S3 and S4, Figure S1C and S1D, respectively). Peak ADMA and SDMA levels were also elevated in non-survivors (2.73 (2.14–3.52) μM vs 1.98 (1.45–2.64) μM, *p* < 0.001 and 2.727 (2.14–3.52) μM vs 1.98 (1.45–2.64) μM), *p* < 0.001), respectively. The area under the receiver operating characteristic curve (SE) for peak ADMA concentration over the study period was 0.705 (0.63–0.78) ) (*p* < 0.001) and for SDMA it was 0.641 (0.56–0.73) (*p* < 0.001). The survival analysis showed that mortality rates increased with increasing concentrations of ADMA and SDMA (Fig. 2c and d, both *p* < 0.001). ADMA concentration in the upper 50% of values was independently associated with mortality in multivariate analysis including SDMA, age, sex, L-arginine and serum creatinine concentration as covariates (hazard ratio 1.82 (1.05–3.14), *p* = 0.033) (Table 2). The ADMA:arginine ratio displayed a similar pattern in the time course studied, with an increased ratio seen on day 3 (*p* = 0.004) and a similar trend on day 7 (*p* = 0.20). (Additional file 1: Figure S1E).

**Table 2:** Asymmetric and symmetric dimethylarginine, L-Arginine and mortality - Cox regression

	Univariate	*P* value	Multivariate	*P* value
ADMA	3.3 (2.0–5.4)	< 0.001	1.82 (1.05–3.14)	0.033
SDMA	2.25 (1.35–3.74)	< 0.001	1.75 (0.95–3.20)	0.071
Age	2.24 (1.36–3.70)	0.002	1.80 (1.07–3.04)	0.027
Female sex	1.088 (0.66–1.79)	0.739	1.20 (0.71–2.05)	0.499
L-Arginine	1.316 (0.80–2.2)	0.283	1.07 (0.60–1.90)	0.812
Creatinine	2.13 (1.28–3.36)	0.004	1.56 (0.87–2.78)	0.14

Patients in the upper two quartiles of L-arginine, asymmetric dimethylarginine (ADMA) and symmetric dimethylarginine (SDMA) concentrations were compared to patients in the lower quartiles. Patients in the upper quartile of age and serum creatinine were compared to those in the lowest three quartiles

### The impact of the rs805305 and eight DDAH1 SNP polymorphisms on outcome in septic shock

**Fig. 3:**
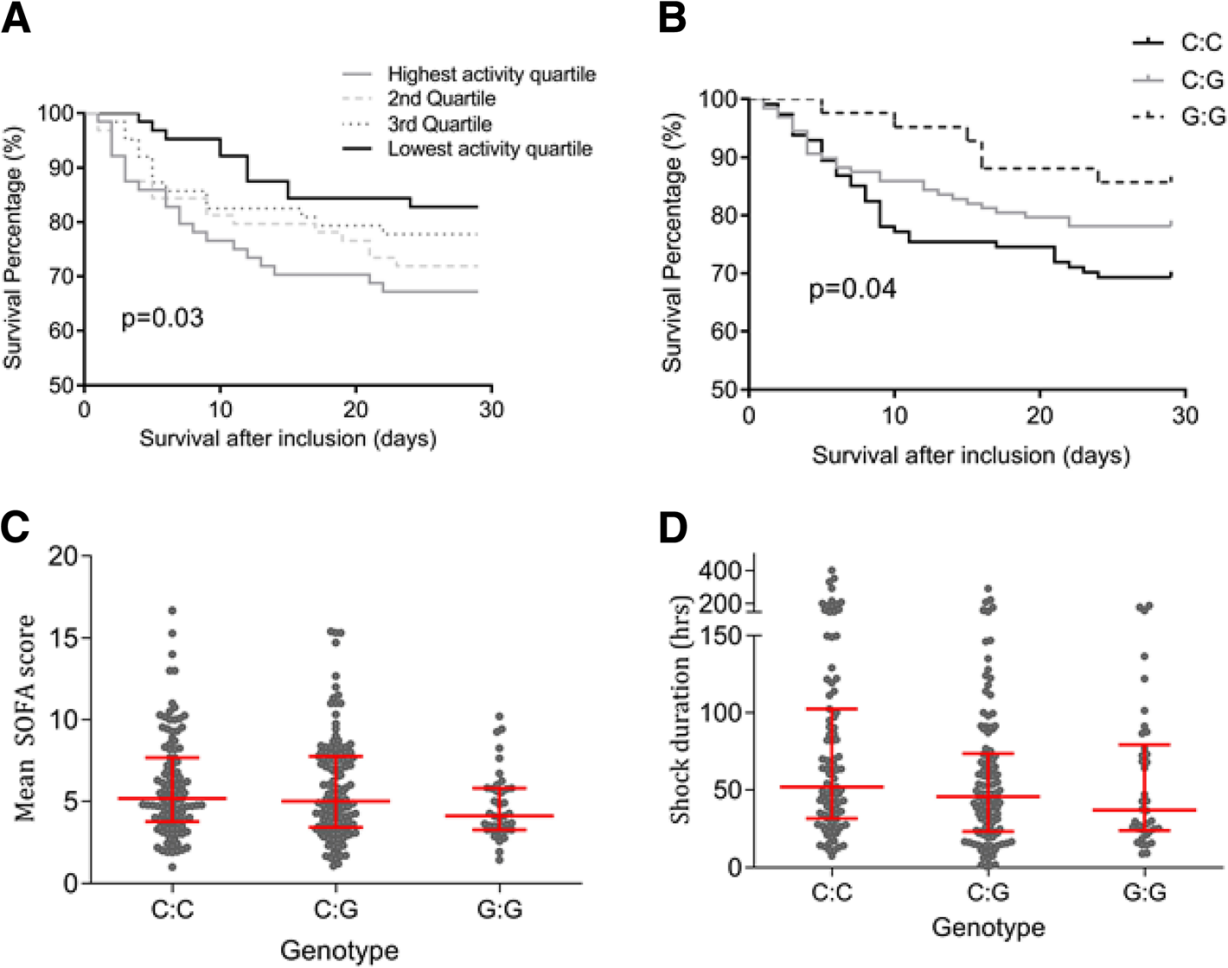
Associations between the rs805305 SNP of the dimethylarginine dimethylaminohydrolase (DDAH)2 promoter region and DDAH activity and outcome in septic shock. **a** Lower DDAH activity is associated with improved outcome in septic shock. Asymmetric dimethylarginine (ADMA):symmetric dimethylarginine (SDMA) ratio was employed as a marker of DDAH activity. Black line - patients with the lowest activity, dotted grey line - 2nd quartile, dashed grey line - 3rd Quartile, solid grey line – highest-activity quartile. The rs805305 SNP of the promoter region of the DDAH2 gene was sequenced in 284 participants in the VANISH trial. **b** Kaplan-Meier analysis of the impact of rs805305 SNP on 28-day survival in septic shock. The presence of the rare G:G homozygote (13.9%) was associated with improved survival compared to the common C:C homozygote (44.7), *p* = 0.02. Mean Sequential Organ Failure Assessment (SOFA) score (**c**) and shock duration (**d**) were lower in patients expressing the less common G:G genotype than the common C:C expression pattern, *p* = 0.04 and p = 0.02, respectively (analysis of variance)

The plasma ADMA concentration was corrected for SDMA concentration as a marker of global DDAH activity. Lower DDAH activity was associated with improved survival on log rank analysis (*p* = 0.033) (Fig. 3a).

The rs805305 SNP of the DDAH2 promoter region was genotyped. The C:C genotype was seen in 128 (44.7%); the C:G heterozygote in 112 (39.1%) and the homozygote G:G haplotype in 40 (13.9%) The G minor allele of the rs805305 SNP was associated with significantly lower 28-day mortality (31% in the common wild-type C:C genotype, 22% in the C:G heterozygotes and 15% in the less common G:G group, *p* = 0.02) (Fig. 3b). Mean Sequential Organ Failure Assessment (SOFA) score during the study period was significantly reduced in patients with the G:G genotype (median (IQR) 4.1 (3.25–5.8)) compared to the heterogozygotes (5.0 (3.4–7.2)) and the common C:C genotype (5.2 (3.8–7.7), *p* = 0.04) (Fig. 3c). ANOVA revealed that duration of shock was also reduced in the G:G genotype (37 (23–79) h) compared to 46 (23–76) h in C:G heterozygotes and 53 (32–109) h in C:C genotype patients (*p* = 0.02) (Fig. 3d). Hospital length of stay displayed a similar pattern, with length of stay of 15.0 (11.0–47.0), 17 (8.25–35.0) and 19 (8.75–40.5) days in the G:G, C:G and C:C groups respectively, although this trend was not statistically significant (Additional file 1: Figure S2). The plasma ADMA:SDMA ratio was also examined in patients with each of the three genotypes of the rs805305 SNP. In the less common G:G homozygotes the median (IQR) ADMA:SDMA ratio was 1.0 (0.69–1.96), in the C:G heterozygotes it was 0.68 (0.51–0.95) and in the common C:C genotype it was 0.64 (0.40–0.91) (*p* = 0.004) (Additional file 1: Figure S3). Homozygotes for the less common DDAH1 SNPs genotyped were found in approximately 1% of the study population. No association with outcome was observed when the risk of death for those expressing the common homozygote genotype was compared to a combined group of heterozygotes and rare homozygotes of each SNP (Additional file 1: Table S5).

## Discussion

This study validates in septic shock the observation made in heterogeneous groups with sepsis that elevated plasma ADMA concentration is associated with outcome. In addition, for the first time, we make the observation that a promoter polymorphism of the *DDAH2* gene, rs805305 is associated with reduced DDAH activity, and in turn improved outcomes in septic shock.

The role of NO in the progression of the inflammatory response in patients with sepsis is complex, with essential upregulation of NO production facilitating appropriate vascular, cardiac and immune responses to pathogenic invasion. However, dysregulation of NO synthesis is a cardinal feature of septic shock and understanding how endogenous inhibitors of NOS enzymes and the genes that regulate them contribute to outcome in sepsis offers the potential for risk stratification and therapeutic development in an area of considerable unmet clinical need.

This is the largest study undertaken to date of methylarginine and L-arginine flux in septic shock and builds on previous work associating ADMA concentrations with outcome. By including only patients with established septic shock in this study, we were able to limit the heterogeneity seen in other studies in which sepsis and severe sepsis diagnoses were also included.

Our findings confirm the observations of a meta-analysis of 192 patients with sepsis, which concluded that L-arginine concentration is significantly reduced in the first week of a septic insult [40], which may be an important anti-inflammatory mechanism and increases the impact of competitive inhibitors of NOS on NO synthesis. We also validated the observation from heterogeneous sepsis cohorts [19, 21, 41] that in sepsis, both plasma ADMA and SDMA are associated with poor outcome, with similar degrees of sensitivity and specificity*.* The relationship between arginine and ADMA concentrations has been proposed as a marker of NOS activity in vivo [23]. Increased ADMA concentrations coupled with reductions in arginine availability in sepsis change the relationship between substrate and competitive inhibitor concentrations leading to reductions in NO production. In this study we found that survivors may have had lower NOS activity after day 3 than those patients that did not survive to discharge from the ICU. This may be consistent with reduced severity of NO-mediated inflammatory response, and is predominantly driven by reductions in plasma L-arginine concentrations. The factors that regulate L-arginine availability are important regulators of the inflammatory response and merit further exploration in septic shock.

These data suggest for the first time in a robust human population with septic shock that an increased ADMA:SDMA ratio is associated with increased survival. This builds on animal and human data suggesting that the plasma ADMA:SDMA ratio may be a better reflection of DDAH enzyme activity than ADMA concentration alone [42, 43]. This reflects the observation that using the circulating ADMA concentration as a marker of DDAH activity is confounded by changes in protein turnover and renal function in acute disease states. Correcting ADMA for SDMA concentration may eliminate this confounder because SDMA handling will be similarly affected by protein turnover and renal failure. Since it is not metabolised by DDAH isoforms, any changes in the relative proportions of ADMA and SDMA is likely to be driven by changes in DDAH activity.

This study shows that in those patients with lower DDAH activity, the resultant increased intracellular ADMA concentrations may confer a survival advantage during an acute disease process such as sepsis by modulating the acute production of excess NO. This is in contrast to plasma ADMA measurement alone and also may reflect a different physiological effect of ADMA in sepsis to that seen in chronic disease states where persistent elevation of intracellular ADMA may be deleterious [44–46]. Previous transgenic animal models have suggested that global and macrophage-specific knockout DDAH2 is associated with poor outcomes in murine models of sepsis [35]. This contrasts with the observations in this study and may reflect the difference between the complete abolition of DDAH2 activity seen in knockout models compared to the modulation of enzyme activity typically seen with functional polymorphisms.

The rs805305 SNP has been examined in pilot studies in sepsis and been associated with plasma ADMA and inflammatory cytokine expression [19, 21, 41]. It has also been associated with the development of hypertension in other populations [47]. Here we show for the first time that the rs805305 SNP is associated with reduced DDAH activity and survival in septic shock. This small study of a genetic association with outcome in septic shock offers insights into the role of endogenous regulators of NO synthesis. Future work will focus on validating this observation and understanding the potential for DDAH2 modulation as therapy in septic shock.

Limitations of this study include a possible bias introduced by using patients included in a randomised controlled trial. The use of results, plasma and DNA from the VANISH trial enabled high-quality data collection in multiple centres that does, however, confer a significant advantage in translational studies of this kind over small, single-centre observational studies. The combination of approaches utilised in this study gives confidence in its conclusions; however, further validation of the role of the rs805305 polymorphism in septic shock in a further data set would be valuable.

Future work should focus on the potential effect of therapeutic modulation of DDAH activity as a whole and DDAH2 specifically merits exploration in septic shock, with the ADMA:SDMA ratio offering an attractive marker of therapeutic efficacy [31].

## Conclusions

In conclusion, this study is the largest yet conducted exploring the associations between DDAH activity and outcome in septic shock. We confirmed the observation that both plasma ADMA and SDMA are associated with outcome. We showed for the first time using the ADMA:SDMA ratio that reduced DDAH activity is associated with improved survival and that the rs805305 SNP of the DDAH2 promoter region is associated with both reduced DDAH activity and improved survival.

## Additional file

Additional file 1: **Table S1.** Median (IQR) peak concentrations of L-arginine, ADMA and SDMA (μM) in patients with septic shock categorised by treatment arm in the VANISH trial; *p* value for Kruskall-Wallis test, no intergroup differences detected on Dunn’s multiple comparison testing. **Table S2.** Pattern of plasma L-arginine concentrations over the first 7 days of the VANISH trial in survivors and non-survivors. Median (IQR) concentrations (μM). **Table S3.** Pattern of plasma ADMA concentrations over the first 7 days of the VANISH trial in survivors and non-survivors. Median (IQR) concentrations (μM). **Table S4.** Pattern of plasma SDMA concentrations over the first 7 days of the VANISH trial in survivors and non-survivors. Median (IQR) concentrations (μM). **Table S5.** Relationship between eight intronic SNPs of DDAH1 and mortality in septic shock**.** The prevalence of the rare genotype in each of the eight DDAH1 SNPs was noted. The odds ratio of death at 28 days after study inclusion was calculated for the common homozygotes against the combined heterozygote and rare homozygote populations. No significant differences in outcome were observed between the groups expressing the less common alleles and the common homozygotes. **Figure S1.** Plasma concentrations of L-arginine (**A**), ADMA (**B**) and SDMA (**C**) in septic shock and association with survival. Plasma from 249 patients with septic shock enrolled in the VANISH trial was collected collection on inclusion into the trial, which was prior to the start of vasopressor therapy (Day 0), and on days 3, 5 and 7 of the study. Dot plot (with median (IQR) overlay in black (survivors) or red (non-survivors)) comparing plasma concentrations on study inclusion and on days 3, 5 and 7 after study inclusion in survivors and non-survivors at 28 days after admission with septic shock. Median (IQR) plasma L-arginine concentrations were similar in non-survivors at each time point. Plasma ADMA and SDMA concentrations were higher in non-survivors at each time point. **D** Plasma SDMA concentration changes over the first 7 days of inclusion in the VANISH trial, notation describes the number of samples available for analysis at each time point. Median and interquartile range plotted in red. **E** Dot plot (with median (IQR) overlay in black (survivors) or red (non-survivors)) comparing plasma ADMA:L-arginine concentrations on study inclusion and on days 3, 5 and 7 after study inclusion in survivors and non-survivors at 28 days after admission with septic shock. Median (IQR) plasma ADMA:L-arginine concentrations appeared higher in non-survivors at days 3,5 and 7. **Figure S2.** Association between the rs805305 SNP of the DDAH2 promoter region and outcome in septic shock. Presence of the rare G:G homozygote was associated with a trend towards reduced hospital length of stay but was not statistically significant. **Figure S3.** Association between the rs805305 SNP of the DDAH2 promoter region and DDAH activity and outcome in septic shock. Plasma ADMA:SDMA ratio was elevated in the rare G:G genotype compared to the common C:C expression pattern, *p* = 0.004. (DOCX 3485 kb)

## Abbreviations

ADMA: Asymmetric dimethylarginine
ANOVA: Analysis of variance
DDAH: Dimethylarginine dimethylaminohydrolase
IQR: Interquartile range
KASP: Kompetitive allele specific
LNMMA: Monomethylarginine
MA: Methylarginine
NO: Nitric oxide
NOS: Nitric oxide synthase
SDMA: Symmetric dimethylarginine
SNP: Single nucleotide polymorphism
SOFA: Sequential Organ Failure Assessment
VANISH: Vasopressin versus noradrenaline as initial therapy in septic shock
