# Supplementary material for: Evidence for a protective role for the rs805305 single nucleotide polymorphism of dimethylarginine dimethylaminohydrolase 2 (DDAH2) in septic shock through the regulation of DDAH activity
Source: Crit Care. 2018 Dec 11;22:336. doi: 10.1186/s13054-018-2277-5 (PMC6288902; doi:10.1186/s13054-018-2277-5)
Supplement: Supplementary file 1 — Table S1. Median (IQR) peak concentrations of L-arginine, ADMA and SDMA (μM) in patients with septic shock categorised by treatment arm in the VANISH trial; p value for Kruskall-Wallis test, no intergroup differences detected on Dunn’s multiple comparison testing. Table S2. Pattern of plasma L-arginine concentrations over the first 7 days of the VANISH trial in survivors and non-survivors. Median (IQR) concentrations (μM). Table S3. Pattern of plasma ADMA concentrations over the first 7 days of the VANISH trial in survivors and non-survivors. Median (IQR) concentrations (μM). Table S4. Pattern of plasma SDMA concentrations over the first 7 days of the VANISH trial in survivors and non-survivors. Median (IQR) concentrations (μM). Table S5. Relationship between eight intronic SNPs of DDAH1 and mortality in septic shock. The prevalence of the rare genotype in each of the eight DDAH1 SNPs was noted. The odds ratio of death at 28 days after study inclusion was calculated for the common homozygotes against the combined heterozygote and rare homozygote populations. No significant differences in outcome were observed between the groups expressing the less common alleles and the common homozygotes. Figure S1. Plasma concentrations of L-arginine (A), ADMA (B) and SDMA (C) in septic shock and association with survival. Plasma from 249 patients with septic shock enrolled in the VANISH trial was collected collection on inclusion into the trial, which was prior to the start of vasopressor therapy (Day 0), and on days 3, 5 and 7 of the study. Dot plot (with median (IQR) overlay in black (survivors) or red (non-survivors)) comparing plasma concentrations on study inclusion and on days 3, 5 and 7 after study inclusion in survivors and non-survivors at 28 days after admission with septic shock. Median (IQR) plasma L-arginine concentrations were similar in non-survivors at each time point. Plasma ADMA and SDMA concentrations were higher in non-survivors at each time point. D Plasma SD [file 13054_2018_2277_MOESM1_ESM.docx]

Evidence for a protective role for the rs805305 single nucleotide polymorphism of dimethylarginine dimethylaminohydrolase 2(DDAH2) in septic shock through the regulation of DDAH activity.

**Short Title:** Lambden. rs805305, DDAH activity and survival in septic shock

**Authors:** Simon Lambden FRCA^$^, James Tomlinson PhD^+^, Sophie Piper PhD^+^, Anthony C Gordon MD^#1^, James Leiper PhD*^1^

Affiliations

^$^ Department of Medicine, university of Cambridge, Addenbrooke’s Hospital, Cambridge, CB2OQQ

* Institute of Cardiovascular and Medical Sciences, University of Glasgow, University Avenue, Glasgow G12 8QQ, MRC London Institute of Medical Sciences, Hammersmith Hospital Campus, Du Cane Road, London, W12 0NN

^+^ MRC London Institute of Medical Sciences, Hammersmith Hospital Campus, Du Cane Road, London, W12 0NN

^#^ Section of Anaesthetics, Pain Medicine and Intensive Care, Faculty of Medicine, Imperial College London, UK

Corresponding Author:

Dr J Leiper

Institute of Cardiovascular and Medical Sciences, University of Glasgow, University Avenue, Glasgow G12 8QQ

1. Joint senior authors. ACG and JL made equal contributions to this study and are listed alphabetically.

James.Leiper@glasgow.ac.uk

Telephone:

Fax:

Supplementary Methods

The clinical population

The sub-population of VANISH trial participants included in this study reflected the VANISH study population as a whole. In order to be eligible, adults were recruited only if they had known or suspected infection, two or more of the systemic inflammatory response syndrome (SIRS) criteria and had failed to respond adequate fluid resuscitation as determined by the treating clinician. Of the 284 patients in this secondary analysis, the median(IQR) age was 65(54-77) and there were 164 men and 120 women. The median(IQR) BMI was 25.7(22.2-31.0) and Acute Physiology And Chronic Health Evaluation (APACHE) II score was 24(19-29). 146 of the patients had been randomised to receive norepinephrine +/- hydrocortisone and 138 were in the vasopressin +/- hydrocortisone group. Patients had received a mean(SD) intravenous fluid volume of 1471(1254)mls prior to study inclusion.

Supplementary results

| Treatment arm | vasopressin + hydrocortisone | vasopressin + placebo | noradrenaline + hydrocortisone | Noradrenaline + placebo | p value |
| --- | --- | --- | --- | --- | --- |
| L-arginine | 41.7  (30.84-55.81) | 34.85  (24.80-56.71) | 38.87  (24.59-60.87) | 34.27  (25.85-51.62) | 0.54 |
| ADMA | 2.21(1.66-3.03 | 2.10(1.51-2.80) | 2.16(1.54-2.85) | 2.23(1.43-2.91) | 0.77 |
| SDMA | 3.45(2.13-5.87) | 3.54(1.94-5.93) | 3.94(1.74-5.72) | 4.42(2.81-6.24) | 0.64 |
| table S1: Median(IQR) peak concentrations of L-arginine, ADMA and SDMA(µM) in patients with septic shock categorised by treatment arm in the VANISH trial,p value for Kruskall Wallis test, no intergroup differences detected on Dunn’s multiple comparison testing. | | | | | |

|  | Inclusion | Day 3 | Day 5 | Day7 |
| --- | --- | --- | --- | --- |
| Survivors | 24.8(17.2-30.6) | 30.7(22.5-42.4) | 32.1(24.7-46.1) | 32.3(23.0-44.9) |
| Non-Survivors | 27.3(16.8-43.9) | 32.1(23.0-42.6) | 38.1(29.5-57.6) | 38.3(26.1-74.5) |
| table S2: Pattern of plasma L-arginine concentrations over the first seven days of the VANISH trial in survivors and non survivors. Median(IQR) concentrations (µM), | | | | |


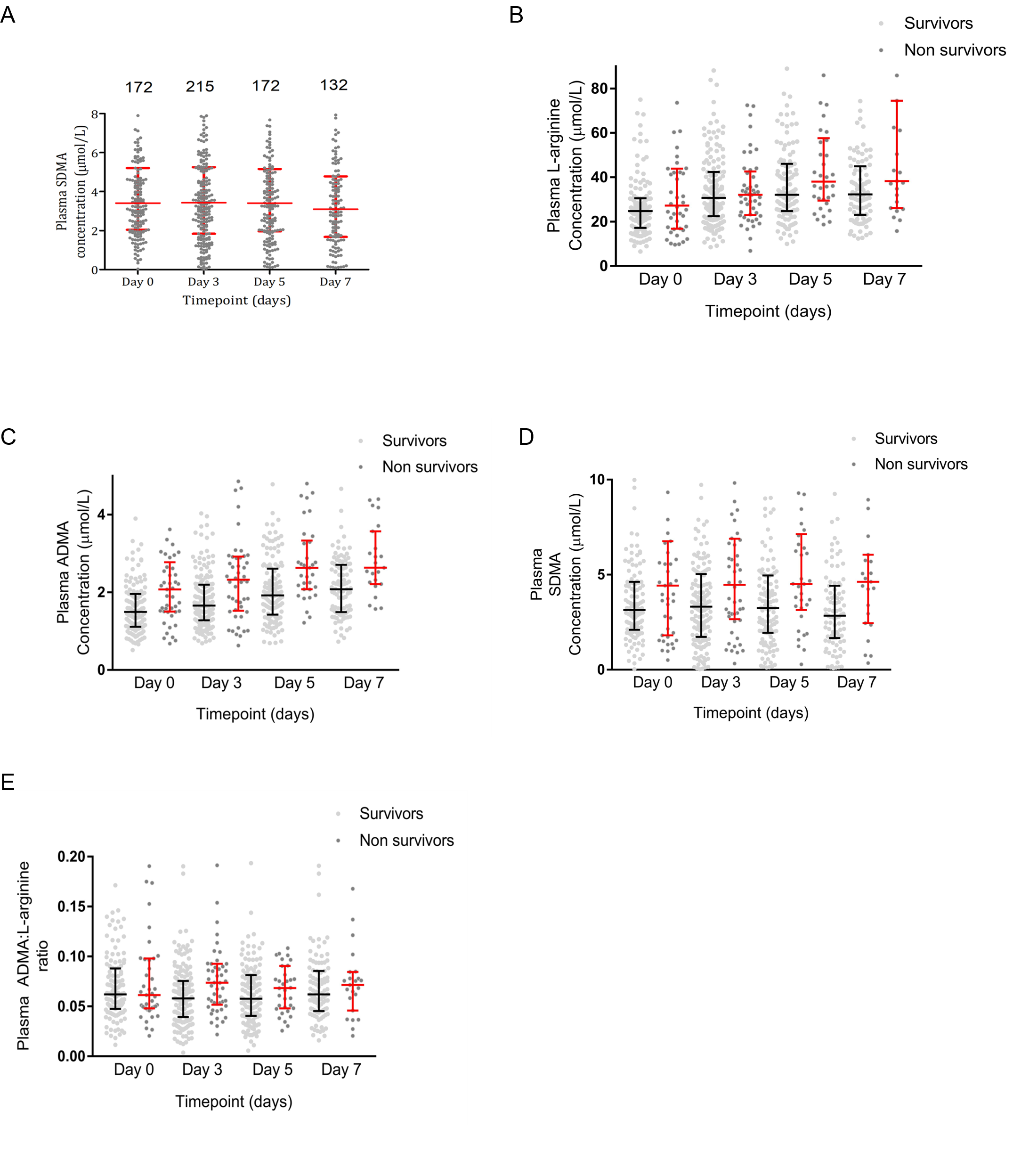


Figure S1: Plasma concentrations of A: L-arginine, B: ADMA and C: SDMA in septic shock and association with survival. Plasma of 249 patients with septic shock enrolled in the VANISH trial were measured in plasma samples collection on inclusion into the trial, which was prior to the start of vasopressor therapy (Day 0), and on days 3, 5 and 7 of the study period. Dot plot (with median(IQR) overlay in black (survivors) or red (non-survivors)) comparing plasma concentrations on study inclusion and on days 3, 5 and 7 after study inclusion in survivors and non-survivors at 28days after admission with septic shock. Median(IQR) plasma L-arginine concentrations were similar in non-survivors at each time point. Plasma ADMA and SDMA concentrations were higher in non-survivors at each time point. D: Plasma SDMA concentration changes over the first seven days of inclusion in the VANISH trial, notation describes the number of samples available for analysis at each time point. Median and interquartile range plotted in red. E: Dot plot (with median(IQR) overlay in black (survivors) or red (non-survivors)) comparing plasma ADMA:L-arginine concentrations on study inclusion and on days 3, 5 and 7 after study inclusion in survivors and non-survivors at 28days after admission with septic shock. Median(IQR) plasma ADMA:L-arginine concentrations appeared higher in non-survivors at days 3,5 and 7.

|  | Inclusion | Day 3 | Day 5 | Day7 |
| --- | --- | --- | --- | --- |
| Survivors | 1.49(1.11-1.96) | 1.66(1.28-2.20) | 1.92(1.43-2.61) | 2.08(1.49-2.71) |
| Non-Survivors | 2.08(1.50-2.78) | 2.33(1.53-2.92) | 2.63(2.08-3.30) | 2.67(2.22-3.57) |
| table S3: Pattern of plasma ADMA concentrations over the first seven days of the VANISH trial in survivors and non survivors. Median(IQR) concentrations (µM). | | | | |

|  | Inclusion | Day 3 | Day 5 | Day7 |
| --- | --- | --- | --- | --- |
| Survivors | 3.15(2.10-4.63) | 3.32(1.73-5.03) | 3.24(1.95-4.96) | 2.85(1.67-4.42) |
| Non-Survivors | 4.43(1.81-6.77) | 4.47(2.66-6.89) | 5.52(3.15-7.13) | 4.63(2.46-6.05) |
| table S4: Pattern of plasma SDMA concentrations over the first seven days of the VANISH trial in survivors and non survivors. Median(IQR) concentrations (µM). | | | | |


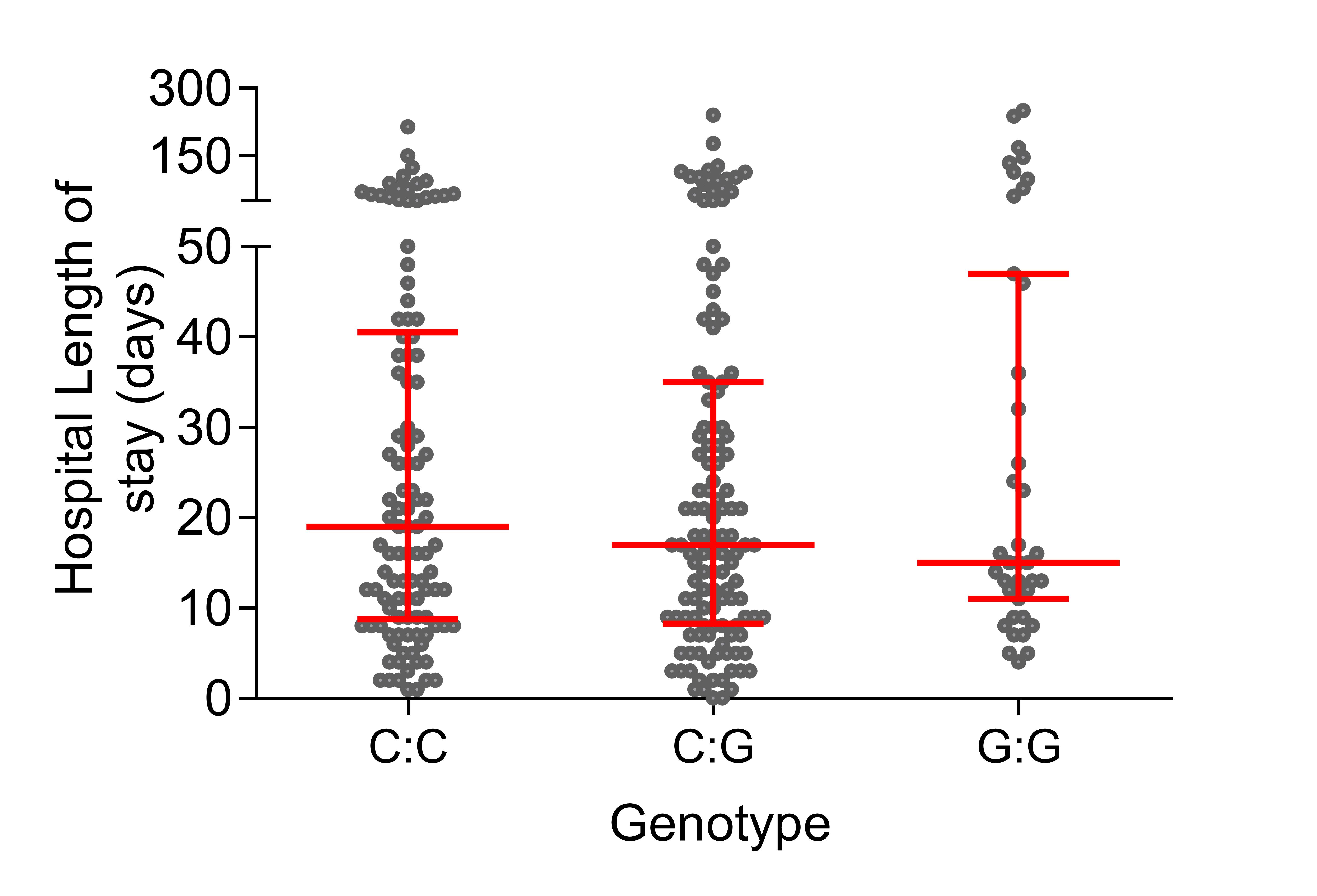


**Figure S2: Associations of the rs805305 SNP of the DDAH2 promoter region with outcome in septic shock.** Presence of the rare G:G homozygote was associated with a trend towards reduced Hospital Length of Stay but was not statistically significant.


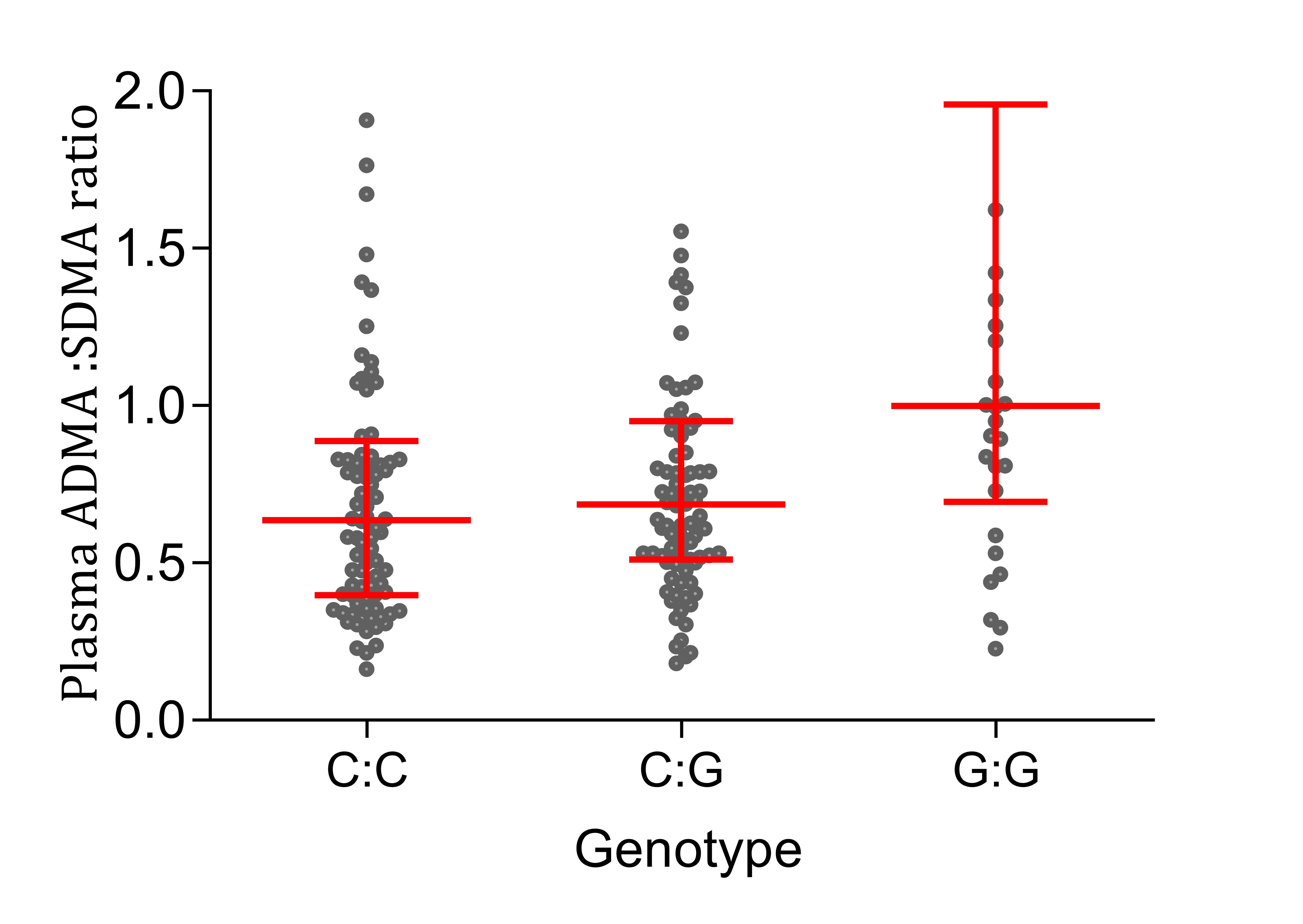


**Figure S3: Associations of the rs805305 SNP of the DDAH2 promoter region with DDAH activity and outcome in septic shock.** Plasma ADMA:SDMA ratio was elevated in the rare G:G genotype compared to the common C:C expression pattern, p=0.004.

| SNP ID | Common homozygote genotype (% prevalence in the population) | Rare homozygote genotype (% prevalence in the population) | p value |
| --- | --- | --- | --- |
| rs1524001 | G:G (86.6) | A:A (1.2) | 0.682 |
| rs7531068 | A:A (86.6) | C:C(1.4) | 0.839 |
| rs10782552 | A:A (86.7) | T:T (1.4) | 0.839 |
| rs897255 | C:C (88.3) | T:T (1.4) | 0.678 |
| rs72726326 | A:A (89.1) | G:G (0.7) | 0.65 |
| rs6576775 | T:T (87.7) | C:C (1.1) | 1.0 |
| rs1378226 | G:G (87.1) | A:A (1.4) | 0.41 |
| rs6682848 | C:C (87.6) | A:A (1.4) | 0.83 |
| **Table S5: Relationship between eight intronic SNPs of DDAH1 with mortality in septic shock.** The prevalence of the rare genotype in each of the eight DDAH1 SNPs was noted. The odds ratio of death at 28 days after study inclusion was calculated for the common homozygotes against the combined heterozygote and rare homozygote populations. No significant differences in outcome were observed between the groups expressing the less common alleles and the common homozygotes. | | | |
